# The sustentaculum tali screw fixation for the treatment of Sanders type II calcaneal fracture: A finite element analysis

**DOI:** 10.12669/pjms.305.5301

**Published:** 2014

**Authors:** Qing-Jiang Pang, Xiao Yu, Zong-Hui Guo

**Affiliations:** 1 Prof. Dr. Qing-Jiang Pang, PhD, Department of Orthopedics Center, Ningbo No.2 Hospital, Ningbo, Zhejiang, China.; 2 Dr. Xiao Yu, PhD, Department of Orthopedics Center, Ningbo No.2 Hospital, Ningbo, Zhejiang, China.; 3 Dr. Zong-Hui Guo, MD, Department of Orthopedics Center, Ningbo No.2 Hospital, Ningbo, Zhejiang, China.

**Keywords:** Calcaneal fracture; Sustentaculum tali; Screws; Finite element analysis; Biomechanics

## Abstract

***Objective:*** In the surgery of calcaneal fracture, whether the sustentaculum tali screw should always be placed is widely controversial. The aim of this study was to explore the necessity and function of the sustentaculum tali screw placement for the treatment of Sanders type II calcaneal fracture.

***Methods:*** The finite element analysis was used in this study. After the establishment of the finite element model of Sanders type II calcaneal fracture, the two internal fixation simulations were designed. In one model, the AO calcaneal plate was simulated on the lateral side of the calcanues with 7 screws being fixated at different position of the plate. In the other model, the calcaneus was fixated with the same AO calcaneal plate together with an additional screw being infiltrated into the sustentaculum tali. The two models were simulated under the same loading and the displacement of the fracture line and the stress distribution in the two models were calculated respectively.

***Results:*** The maximum principal stress focused on the cortical bone of sustentaculum tali in both the models under the same loading. The displacement of the fracture line, the maximum principal stress of calcaneus and internal fixation system in the model with sustentaculum screw fixation were smaller than that in the model without sustentaculum screw fixation. The stress in the model with sustentaculum screw fixation was more dispersed.

***Conclusions:*** The placement of sustentaculum tali screw is essential for fixation of type II calcaneal fracture to achieve the biomechanical stability.

## INTRODUCTION

Calcaneal fracture is common seen fracture in foot and ankle surgery, which accounts for 60%~65% of the entire tarsal bone fractures.^1^ The improper treatment of calcaneal fracture might lead to the severe calcaneal malunion, which can result in abnormal gait with symptoms and dysfunction of the joints and muscles, eventually leading to the pain and permanent disability. 2

When the calcaneal fracture occurs, the vertical pressure from the talus often causes the calcaneus to be compressed while the posterior articular surface is usually comminuted. However, as the powerful tendons and ligaments in the surrounding of the sustentaculum tali, the displacement of the sustentaculum tali rarely occurs in the calcaneal fracture, which might provide powerful fixation site for the screws to obtain stable fixation in biomechanics.^3^ Up to now, the relative biomechanical research of the fixation of the sustentaculum tali is seldom reported.

In this study, we established the three-dimensional (3D) finite element model of Sanders type II calcaneal fracture and simulated two kinds of fixation methods, namely AO calcaneal plate with an additional sustentaculum tali screw fixation or without sustentaculum tali screw fixation. The aim of this study was to observe the biomechanical effect of the sustentaculum tali screw to the model of calcaneal fracture and explore the necessity and function of the sustentaculum tali screw placement for the treatment of Sanders type II calcaneal fracture.

## METHODS

***General data: ***A 30 year-old healthy female volunteer (height 163cm and weight 60 kg) was recruited as the experimental object. The X-ray examination showed no deformity and damage in the foot.

The equipment and software include: (1) The SOMATOM Definition 4D dual source CT (Siemens Ltd, German); (2) Medical imaging software: Mimics 12.0 (Materialise Ltd, Belgium); (3) Three dimensional optimization software: Geomagic Studio (Rainrrop Ltd, USA); (4) Solid Works 2010 (Dassault Systemes Ltd, USA); (5) The finite element analysis software: ANSYS 13.0 (ANSYS Ltd, USA).

**Experimental method:**

***Data collection: ***The 4D dual source CT was used to scan the volunteer from middle segment of the tibial to the whole part of the foot in neutral position. The slice thickness was 1 mm and the scan speed is 0.4 s / ring. The CT image data of 512×512 matrix was obtained (Dicom format).

***The establishment of 3D model*** *: *The Dicom format data was loaded in the software of Mimics 12.0 to obtain a three-dimensional model of calcaneus. After the optimization of the model, it was loaded to the software of Ansys 13.0 and a calcaneal three-dimensional finite element with 1775784 nodes and 1088931 units could be obtained. The materials in the model were simplified as homogeneous elastic materials. The thickness of the calcaneal cortical bone was set in 2 mm and the homogeneous cancellous bone inside.

The model of calcaneus was then loaded into the Solidworks 2010 to simulate the Sanders type II calcaneal fracture according to the definition of the Sanders classification. In order to reflect the stress distribution of the calcaneus between the fracture line better, the fracture line was replaced by the soft material. When the model was loaded by the external force, the displacement of the soft material could reflect the displacement of the implants. 

The geometric parameters of the AO lateral calcaneal anatomical plate and screws were loaded into the Solidworks 2010 and two kinds of internal fixation model were established according to the design of the experiment. One kind was that the plate was placed at the lateral side of the calcaneus and 7 cancellous screws were respectively fixated to the plate from different directions, among which two were at the body the plate under the posterior articular facet of the calcanues; two were at the back of the plate; one was at the bottom of the plate and other two were in the front of the plate. The other kind was that with the same fixation method as above, an additional cortical screw was placed into the sustentaculum tali from the bottom of the posterior articular surface of the plate.^3^ The elastic constants of the different materials were set as shown in Table-I.

**Table-I:** The elastic constants of the different materials

***Materials***	***Modulus of elasticity(*** ***Mpa*** ***)***	***Poisson ratio***
Cortical bone	7300	0.3
Cancellous bone	100	0.3
Titanium plate and screws	200000	0.28
Fracture line	5	0.4

***The loading of the external force: ***In this study, we set the lowest contact point of the calcaneocuboid articular surface and the calcaneus with the ground as the constraint point. The loading direction was set from top to bottom in the posterior and middle subtalar articular surface, while the reverse direction was set in the calcaneal tuberosity. The tensile force caused by the traction of the intrinsic muscle that originates from the calcaneus could be offset according to the principle of the synthesis and decomposition of the force.^4^ The simulative set of the load in the neutral position of the calcaneus in 3D model could be seen in Table-II.

**Table-II:** The load and nodes in each surface in the neutral position of the calcaneus

***Articular surface or Contact surface ***	***Node***	***Load(*** ***N*** ***)***
Contact surface between the Achilles tendon and ground	1620	Constraint
Calcaneocuboid articular surface	950	Constraint
Middle subtalar articular surface	1348	200
Posterior subtalar articular surface	1682	420
Calcaneal tuberosity	1563	-300

## RESULTS

**Fig.1:**
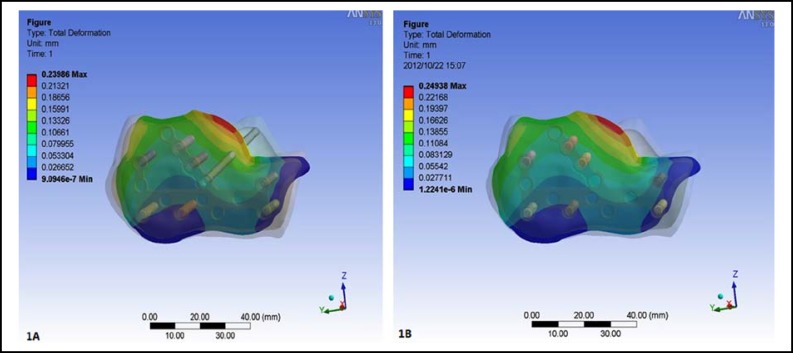
The displacement of the fracture line. **A.** The minimum displacement of the fracture line in the model with sustentaculum tali screw was 9.0946×10^-7^ mm and the maximum displacement was 0.23986 mm. **B.** The minimum displacement in the model without sustentaculum tali screw was 1.2241×10^-6^ mm and the maximum displacement was 0.24938mm

***The displacement of the fracture line: ***After loading, the minimum displacement of the fracture line in the internal fixation model with sustentaculum tali screw was 9.0946×10^-7^ mm and the maximum displacement was 0.23986 mm, while the minimum displacement in the model without sustentaculum tali screw was 1.2241×10^-6^ mm and the maximum displacement was 0.24938mm (Fig.1).

**Fig.2:**
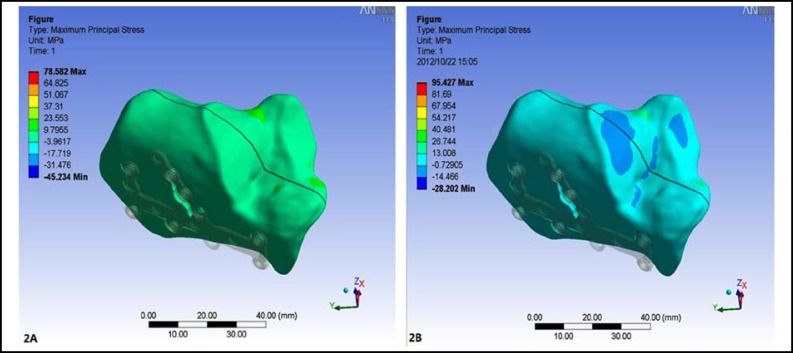
The stress distribution in the calcaneus. **A.** The maximum stress in the model with sustentaculum tali screw was 78.582 MPa, which was mainly concentrated on the cortical bone around the sustentaculum tali. **B.** The maximum stress in the model without sustentaculum tali screw was 95.427 MPa, which was also mainly concentrated on the cortical bone around the sustentaculum tali

***The stress distribution in the calcaneus: ***After loading, the maximum stress in the calcaneal model with sustentaculum tali screw was 78.582 MPa, which was mainly concentrated on the cortical bone around the sustentaculum tali. While the maximum stress in the model without sustentaculum tali screw was 95.427 MPa, which was also mainly concentrated on the cortical bone around the sustentaculum tali (Fig.2).

**Fig.3:**
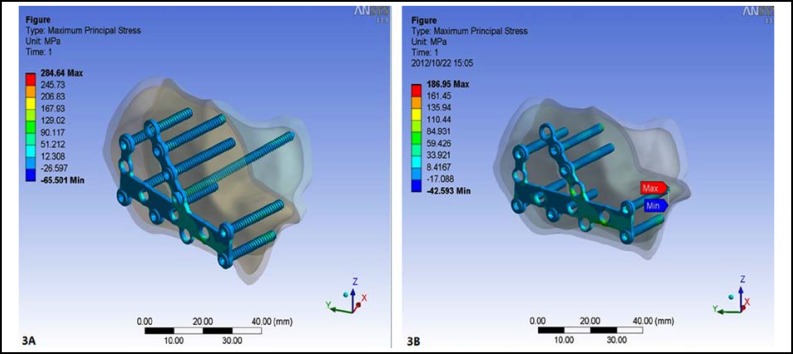
The stress distribution in the implants.** A.** The maximum stress in the implants with sustentaculum tali screw was 284.64 MPa, which was mainly concentrated in the middle part of the plate. **B.** The maximum stress in the implants without sustentaculum tali screw was 186.95 MPa, which was mainly concentrated in the posterior subtalar surface around the tail of the cancellous screws

***The stress distribution in the plate and screws: ***After loading, the maximum stress in the implants with sustentaculum tali screw was284.64 MPa, which was mainly concentrated in the middle part of the plate. While the maximum stress in the implants without sustentaculum tali screw was 186.95 MPa, which was mainly concentrated in the posterior subtalar surface around the tail of the cancellous screws (Fig.3).

As for the analysis of the sustentaculum tali screw, the minimum displacement of the sustentaculum tali screw was 0.040659mm and the maximum displacement was 0.15642mm. The maximum stress in the sustentaculum tali screw was 142.92 MPa, which was mainly concentrated in the middle part of the screw.

## DISCUSSION

***The establishment of the*** ***3D finite element*** ***model of internal fixation of the calcaneus: ***Because of the complicated and irregular anatomical structure of the calcaneus and its surrounding soft tissues, the biomechanical research on the corpse specimens of calcaneus is difficult to be carried on. However, as the special ability of high accuracy simulation and speedy calculation of complex shapes, loading and material properties, the finite element analysis (FEA) is always substituted for the corpse specimen biomechanical research.^5^^,^^6^ In the study, the Dicom format data from CT scan of the calcaneus was loaded in the software of Mimics12.0 to obtain the initial 3D model of the calcaneus, then the software of solidworks 2010 could be used to cleave the model according to the Sanders’ classification to achieve the model of Sanders type II calcaneal fracture. The software Ansys 13.0 can simulate the operations and assign the physical properties to the implants. After loading, the calculation could be carried on to work out the displacement of the fracture line, the stress distribution in the bones and implants. However, as the limitation of the finite element analysis, the mechanical properties of materials are defined as continuous and homogeneous, isotropic, this assumption is slightly different from the real situation of the calcaneus itself. ^7^^,^^8^

***The biomechanical effect of the sustentaculum tali screw to the internal fixation of Sanders type*** ***II*** ***calcaneal fracture: ***In order to reflect the displacement and the stress distribution of the fracture line more veritably, the fracture line was replaced and bonded by the soft material with the Modulus of elasticity 5 MPa and Poisson ratio 0.4. The results showed that the minimum displacement of the fracture line in the model with sustentaculum tali screw was 9.0946×10^-7^ mm and the maximum displacement was 0.23986 mm, while the minimum displacement in the model without sustentaculum tali screw was 1.2241×10^-6^ mm and the maximum displacement was 0.24938mm. We only analyzed the maximum data of the results because there might have no significance to analyze the minimum data. The results showed that the displacement of the model with sustentaculum tali screw was smaller than that of the model without sustentaculum tali screw. It testified that the sustentaculum tali screw could provide additional stability to the Sanders type II calcaneal fracture.

According to the biomechanical standpoint, the stress in implants should be decentralized as far as possible to avoid the implant breakage.^9^ The maximum stress in the calcaneal model with sustentaculum tali screw was 78.582 MPa, which was mainly concentrated on the cortical bone around the sustentaculum tali. While the maximum stress in the model without sustentaculum tali screw was 95.427 MPa, which was also mainly concentrated on the cortical bone around the sustentaculum tali. The results showed that the maximum stress of the model with sustentaculum tali screw was smaller than that of the model without sustentaculum tali screw. It suggested that the screw fixation in sustentaculum tali could make the stress relatively decentralize, which was more suitable for fixation in the biomechanical requirement.

At present, the cannulated screw, the cancellous screw and cortical screw could all be chosen for the fixation of sustentaculum tali. In this study, we also analyzed the sustentaculum tali screw alone and found the maximum displacement of the sustentaculum tali screw was 0.15642mm and the maximum stress in the sustentaculum tali screw was 142.92 MPa, which was mainly concentrated in the middle part of the screw. Furthermore, the stress in sustentaculum tali screw will also increase gradually with the increased load in the subtalar articular surface. Therefore, we chose the cortical screw for the fixation of the sustentaculum tali to play the “backbone” role in the fixation of calcaneal fracture.^10^

***The clinical significance of the sustentaculum tali screw: ***In the calcaneal fracture, the main factors that affected the prognosis are the congruity of the articular surface and the fixation of the fractures.^11^ Therefore, how to fixate firmly in the calcaneal fracture is always a hot topic in clinical research. Occasionally, in some comminuted calcaneal fractures, the screws could only provide poor holding forces because of the pultaceous cancellous bone inside in the lateral wall of the calcaneus, which will eventually lead to the failure of the fixation. The sustentaculum tali is one of the most important anatomic marks in the calcaneus. As it was constrained by the joint capsule and surrounding ligaments and tendons, the sustentaculum tali fragment will seldom be displaced from the medial wall of the calcaneus, even if the severe calcaneal fracture occurs.^12^ Therefore, the screw could be placed to the sustentaculum tali from lateral wall of the calcaneus to increase the stability of the implants.^13^ This study also showed the model with sustentaculum tali screw has less displacement of the fracture line and smaller maximum stress in the calcaneus and implants than the model without sustentaculum tali. The sustentaculum tali screw fixation for the treatment of Sanders type II calcaneal fracture is more suitable for the requirement of biomechanics and could be widely used in the clinic.

## Notes

***Acknowledgement:*** This study was supported by a grant from the Social Development Foundation of Ningbo City.(No. 2011C50063).
